# Transparency or restricting gifts? Polish medical students’ opinions about regulating relationships with pharmaceutical sales representatives

**DOI:** 10.1007/s40592-021-00128-2

**Published:** 2021-06-07

**Authors:** Marta Makowska, Emilia Kaczmarek, Marcin Rodzinka

**Affiliations:** 1 Institute of Sociological Sciences and Pedagogy, Warsaw University of Life Sciences, Nowoursynowska 166 St., 02-787 Warsaw, Poland; 2 Faculty of Philosophy, University of Warsaw, Krakowskie Przedmieście 3, 00-927 Warsaw, Poland; 3 Jagiellonian University Medical College, Kraków, Poland

**Keywords:** Pharmaceutical marketing, Legal regulations, Transparency, Medical students, Qualitative research, Bioethics

## Abstract

Relationships between physicians and pharmaceutical sales representatives (PSRs) often create conflicts of interest, not least because of the various benefits received by physicians. Many countries attempt to control pharmaceutical industry marketing strategies through legal regulation, and this is true in Poland where efforts are underway to eliminate any practices that might be considered corrupt in medicine. The present research considered Polish medical students’ opinions about domestic laws restricting doctors’ acceptance of expensive gifts from the industry, the idea of compulsory transparency, and the possibility of introducing a Polish Sunshine Law. A qualitative, focus group-based, interview method was used. Data were gathered from nine focus groups involving 92 medical students from three universities located in major Polish cities. The article presents a classification of opposing student views with regard to the consequences of introducing different legal solutions; this should be useful for policy makers deliberating on how to optimally regulate pharmaceutical marketing. The study’s results are discussed in the context of the public bioethical debate in Poland.

## Introduction

In Poland and other countries, healthcare professionals (HCPs) and health industries interact in many ways, including in research-oriented collaborations and in marketing activities (Fickweiler et al. 2017). It has been well documented that close ties with industry may result in biases in physicians’ decision making that are concordant with industrial interests (Fickweiler et al. 2017; Choudhry et al. 2002; Van Harrison 2003; DeJong et al. 2016; Yeh et al. 2016). Even when practitioners abide by ethical codes, it is not always easy for them to recognize the extent of the influences and pressures to which they are subjected (Sah and Fugh-Berman, 2013). Some studies have shown that HCPs do not usually recognize how gifts from pharmaceutical sales representatives (PSRs) can influence their prescription habits (Fickweiler et al. 2017; Makowska 2017). Also, opinions about the quality of information provided by PSRs are divided (Hodges 1995).

Pharmaceutical companies concentrate their activities relating to the medical community on relationship-based marketing, creating and maintaining long-term relationships with their clients (Berry 1983). Relationships between physicians and the pharmaceutical industry have attracted previous interest from Polish researchers (Makowska 2010; Polak 2011; Makowska 2017). Makowska (2017) asked 397 physicians four questions about various legal aspects of cooperation with companies, and as many as 17.2% of physicians failed to answer any of the questions correctly, and only 21.6% correctly answered all of the questions. Further, the study also showed that the law banning meetings with PSRs during working hours was often violated, and that, as already shown by earlier Polish research (Makowska 2010; Polak 2011), laws limiting the value of gifts were sometimes circumvented.

While the mainstream of research in this field focuses on the relationship between the industry and physicians, it should not be forgotten that the industry also targets medical students: studies from different countries show that medical students are often exposed to pharmaceutical industry marketing activities even though they are unable to make prescribing decisions (Fitz et al. 2007; Austad et al. 2013). To date, these relationships have received much less attention than those involving physicians (Sierles et al. 2005; Carmody and Mansfield 2010; Austad et al. 2011) despite the fact that research on students is highly important because the socialization which occurs during students’ time at medical school plays an important role in forming the attitudes that they will take with them into their future professional practice.

As in other countries, Polish medical students have contacts with PSRs, although the nature of these is yet to be thoroughly studied. Nevertheless, one study^1^ found that almost 60% of final year students at a medical school in Warsaw had received a gift from a pharmaceutical company, and almost 66% had participated in educational meetings organized by a pharmaceutical company (Makowska et al. 2017). Despite this, there is a lack of regulations in Polish medical schools regarding university, lecturer, and student cooperation with the pharmaceutical industry. In the wider European context, Poland is no exception here. For example, it has been found that only 24% of French medical schools have either any type of conflict of interest (COI) policy or include COI in the curriculum (Scheffer et al. 2017), and in Germany this proportion is only 5.3% (Grabitz et al. 2019).

The present research aimed to ascertain the opinions of Polish medical students concerning two legal solutions to the potential problems which arise from physician—industry cooperation. The first forbids PSRs from giving physicians gifts of more than PLN 100 (27 USD/24 EUR) in value. Such a ban is in force under current Polish pharmaceutical law (Polish Journal of Laws of 2001 No. 126, item 1381, article 58^2^). The second legal solution considered was the introduction of transparency, such as the mandatory disclosure of all benefits received from PSRs on a governmental web site. Policies of this type are usually called Sunshine Laws—for example, the U.S. Physician Payments Sunshine Act. It should be noted that currently only voluntary codes regarding transparency apply in Poland (INFARMA 2015; Polski Związek Pracodawców Przemysłu Farmaceutycznego 2017).

While there is growing evidence relating to HCPs’ opinions regarding pharmaceutical marketing and its regulation, investigations of medical students’ opinions are lacking, and the present study aimed to at least partially fill this gap.

## Materials and methods

### Study design

A qualitative, focus group-based, interview method was used. Following Flick’s (2007) recommendations for conducting focus groups, a social constructivist perspective was adopted. Nine focus groups were assembled. The discussion sequence^3^ was the same in each group and consisted of three stages: (1) an introductory stage—giving participants the chance to get to know each other; (2) a discussion of socialization to the medical profession, and; (3) a discussion of relationships between pharmaceutical companies and medical students. Two articles based on the focus group interviews have previously been published (Kaczmarek and Makowska 2020; Makowska 2021), but neither of these concerns legal regulation of pharmaceutical marketing.

According to Braun and Clark, the term “data set refers to all the data from the corpus that are being used for a particular analysis.” (p. 79). The analysis described in this article used a data set, assembled from the focus group interviews, which related to medical students’ opinions about the previously mentioned legal solutions for regulating pharmaceutical marketing.

### Sampling

As mentioned previously, nine focus groups were assembled, with an average of 10 participants in each (range 7–13 people). The main requirement of respondents was that they were a student in their second year or above at a medical university in three Polish cities (Gdansk, Warsaw, and Krakow: the three main cities in the north, center and south of Poland respectively). Ninety-two medical students took part in the study. Only basic demographic information about participants was obtained: their gender and year of study. There were 52 females and 40 males, seven students in their second year of study, 41 in their third year, 20 in their fourth, 11 in their fifth, and 13 in their sixth.^4^ Each focus group contained a mixture of students in different years and of different genders.

### Recruitment

All participants were invited to participate by a specialized recruitment company so that they remained anonymous to the researchers. Prior to participation, all participants received information saying that the research would be about drug manufacturers’ presence in medical universities and its possible influence on students’ socialization to the medical profession. No further specific study objectives were presented to participants. Each participant received remuneration of 150 PLN (35 EUR). Ethics committee approval was not sought since this was not required by the institution funding the study, but all participants gave their informed consent before participating.

### Data collection

Focus group sessions were held at professional focus group studios. All sessions were moderated by the study’s principal investigator (MM). Sessions lasted approximately two hours and were conducted using a script containing open questions. All sessions were recorded on video and a dictaphone, and professionally transcribed by people not involved in the study. All transcriptions were checked by the PI to ensure that they corresponded with recordings. No attempt was made to link any utterances with particular participants to preserve confidentiality.

### Data analysis and handling

The data collected were examined using thematic analysis, which is particularly well suited to handling focus group data. Thematic analysis was performed in the phases recommended by Braun and Clark (2006): (1) familiarization with the data; (2) generation of initial codes (from the corpus of data, a data set relevant to the currently considered issues was identified, and it was decided that this was worthy of closer examination); (3) a search for themes; (4) a review of the themes identified; (5) definition and naming of themes; (6) the production of a report. In our analysis we distinguished four main themes: (1) lack of education on the issues involved; (2) the impact that legally restricting the value of gifts has on doctors’ integrity and patients’ trust; (3) the impact of transparency laws on doctors’ integrity and patients’ trust; (4) barriers to introducing a sunshine law in Poland. Below, we describe these four themes in detail, and include descriptions of opposing student views.

## Results

### Issues surrounding relationships with PSRs, and their legal basis, are rarely raised during students’ studies

The first finding of the study suggested that issues surrounding relationships between physicians and PSRs are rarely, if ever, covered during Polish medical education. Seemingly, most of the students participating in the focus groups only learned about the Polish law restricting the giving of gifts during the group interviews. They were also unfamiliar with other countries’ legal safeguards regarding the transparency of relationships between doctors and pharmaceutical companies. Moreover, when discussing sunshine solutions, none of the students mentioned the voluntary code in force in Poland. When asked if these topics were discussed during university classes, the vast majority in all groups said that they were not. Only a few students said that such issues might have been mentioned during lectures on medical law or pharmacology.

In many groups there was a clear demand for people to be better informed about the Polish law banning excessive gifts—not only the students themselves and physicians, but also patients in order to prevent suspicions as to what occurs behind a closed door when a PSR is in a doctor’s office.

*F1*^5^* (III) I mean, it is poor …widespread*.

*M1(VI) I think no one knows about it*.

*F2 (IV) Exactly. None of us knew about it. (…). (Warsaw, GP2)*

When asked if such issues should be covered during studies, most participants said that it would be useful to know what is legal and what is forbidden in relationships with PSRs.

*M1 (VI) It would be good if someone could tell us what this is all about.*

*M2 (VI) What we are allowed to do, and what is not allowed.* (Warsaw, GP2)

One participant said that she did not feel well prepared for her future professional relationships with PSRs, explaining this as follows:

*F (V) But actually, we don't know how to talk to these representatives (…). We learn only by observing other doctors. We use our own assertiveness. Our own conscience. Well, we don't know how to talk to them, how to come to an agreement, or how to make them leave us alone (laughs).*^6^ (Gdańsk, GP2)

Many students met a PSR for the first time during a summer internship in a family doctor's office. They often criticized the fact that PSRs cut into queues of patients and limited doctors’ time with patients. They expressed surprise when informed that, under Polish Ministry of Health regulations (Polish Journal of Laws of 2008 No. 210, item 1327), physicians should not meet with PSRs during their working hours. Also, many participants thought it unlikely that doctors themselves would be familiar with these laws.

*Moderator: Do you think that doctors are aware that these gifts can only have a value up to PLN 100 and that doctors are not allowed to receive representatives during working hours?*

*F (III) No, definitely not. (Warsaw, GP1).*

When asked about the gifts that they knew physicians had received, they did not mention anything above the value of 100 PLN, and therefore they did not report any breaches of the law. Doctors (and sometimes even the students) received small gifts from the pharmaceutical industry (e.g., memory sticks, post-it notes, mugs, pens, educational materials, books, etc.). The only more expensive gifts that appeared in the statements were stethoscopes. Some participants were convinced that physicians had received more expensive gifts in the past.

### Opinions about the impact of legal restrictions on the value of gifts on doctors’ integrity and patients’ trust

During group discussions about limiting the value of doctors’ gifts to PLN 100, students often mentioned the issue of patients’ trust and suspicions about corruption. According to some students, wider dissemination of this law’s existence could improve public opinion about relationships between doctors and the pharmaceutical industry.

Some students emphasized that the law could prevent corruption, because PLN 100 is not enough to bribe anyone. However, students differed in their opinions about PLN 100 being large or small amount. These differences probably depended on their own financial situations. Some of them said that this was a significant amount for them. They also thought that patients would perceive this as a high amount. However, when they took the perspective of a doctor with a long history of practicing, and their earnings, this amount did not appear to be attractive.

*M (III): It is not a lot, as the hourly rate for a doctor working at an emergency department in Radom is PLN 200. *(Warsaw, GP3).

A few participants also criticized the law in terms of PLN 100 being too low a limit. In their opinion, this limited the possibilities of enjoying benefits provided by the pharmaceutical industry. They thought that because the industry has money, physicians should be able to use this money.

There were also statements suggesting that if a representative did not bring anything, or only brought a minor thing, then some physicians would not want to meet them anymore. Therefore, some students thought that the law may also discourage doctors from meeting with PSRs.

*M (IV) Well, I won't feel like I want to meet with him if he doesn't give me anything. (laughs).* (Krakow, GP2)

Rarely, there were students who saw the acceptance of anything from a pharmaceutical company as a threat to a physician’s impartiality. A more popular view was that less valuable gifts, such as those up to a value of PLN 100, would not trigger the need for reciprocity: the desire to repay. Interestingly, students were aware that the reason why physicians are given gifts is to enhance product sales by means of evoking positive associations with respect to a given brand in a doctor’s memory or subconscious.

*F (V): We know that they want to buy us. (…) It is known what they want. They are not guided by the benefits to patients, they are guided by their profit (…)*

*M (VI): But, in the end, every doctor makes their own decision, they have free will and prescribe medication as they think fit. And this is the best for the patient. So, there may be such biases, subconscious actions, but it seems to me that every [doctor] is guided by good. And if the patient says that a drug is too expensive, they are prescribed a cheaper one, not the one who ['s producer] gave us money for a conference.* (Gdańsk, GP2)

Most participants seemed aware that relationships with PSRs are of a marketing nature. At the same time, however, most were convinced that they would not make decisions which harmed their patients because they had received a gift from a certain company. This said, some students admitted that gifts could influence them, for example, if they had to choose between identical products from different companies.

*F (V): Since they do it, it means that it must be effective, but I guess that it is not worse for the patient. [If]…a doctor prescribes this drug, and it is the same medicine as another company’s, but this one [company] is associated with us, I will prescribe theirs.* (Gdańsk, GP2)

What is more, students emphasized that some gifts are strictly educational, that others (e.g., glucometers) are accepted for the benefit of patients, and that, because the Polish healthcare system is underfunded, poverty prevails in healthcare facilities.

*M (IV) (…) it is not pathological (pressure in voice) that someone brings these pens, but the fact that a department is not properly equipped with pens*.

*F (V) Yes (loudly, nodding)*.

*M (IV) I think it (pressure) should arouse controversy that this doctor accepts bribes in the form of pens.* (Warsaw, GP3)

Many students stressed that it would not be worth risking one’s medical opinion or patient trust for PLN 100, but if the amount were larger the temptation might be greater.

*M (V) I can lose my patients, their trust and my good image, so ... gifts ... Well, I will check* [a drug] *(…) before I recommend it to anyone. And how big would a gift have to be for me to risk losing my reputation in the city?*

*F (III) Well, but the point is that these PLN 100 are in the form of a gadget and promotion, not necessarily a bribe. And at this point, if the amount were larger, I can understand that someone could be more willing to be bribed and take a risk ...* (Gdansk, GP1)

On the other hand, there was often a thread in students’ statements to the effect that the PLN 100 limit is artificial because someone had arbitrarily decided that “inappropriate behavior” starts above this threshold. In the opinion of some respondents, it is difficult to set such a limit, which may be different for everyone, and so the law removes a physician’s responsibility to consider whether receiving a gift is moral and whether it may affect their judgment in any way. According to some students, the act formalizes the receipt of gifts and eliminate moral doubts.

*F(III) I mean, I wouldn't despise it because it's a law, it's like I know I can accept a gift. It gives me … moral freedom and peace (laughs).* (Gdansk, GP2)

*F1 (III) It seems to me that this is not a matter (…) of PLN 100 (…), or whether it should be, PLN 200, 300, 500 or 50, but it is a matter of what moral view a person has on this. And it seems to me that morality cannot be changed so easily: that the government will introduce a law according to which PLN 100 is the limit and then I change my way of thinking: “oh good, the government told me that PLN 100 is still cool, that's ok, I'll prescribe”.*

*M1 (III) I think that the very fact of introducing this provision also imposes a need for remorse on a doctor.* (Krakow, GP1)

In a few focus groups, a few students expressed the idea that doctors should not accept gifts at all. While these utterances were usually incidental, in one of the groups there was a strong personality with such views, and the discussion between him and others caused some members of the group to agree that there should be no gifts at all (Krakow, GP3). In this group, it was noted that the law causes a change in perceptions of a bribe, which is redefined as a gift.

*M (IV) This is a bit of a rhetorical question. Why, if we accept something of value, any object, or money of a given value from an ordinary person (…) from each of us, it's a bribe? But if it is from someone in a stronger position, it is a gift? (silence) Mmmm? I mean, can anyone answer that question for me? (…) I would forbid both. The same rules for everyone.* (Krakow, GP3)

There was also a thread in many groups that criticized faulty drafting of the Polish law. There is no set period during which one can accept gifts with a total value of PLN 100. Students easily came up with ideas on how to circumvent the law with respect to this. Most commonly, it was said that because the law refers to a one-time gift, a doctor could be visited several times in a short time and the gifts given would sum to a “significant” amount. Students also speculated that companies could have a deal with third parties and be able to buy expensive items more cheaply – just up to the PLN 100 limit. There was also the idea that since the law only refers to a company offering gifts up to the value of PLN 100, PSRs could supplement this to buy something more expensive.

*F (II) So s/he can receive 3 gifts for PLN 100*. (Gdansk, GP3).

*M (III) Let's take this chair, assuming it costs PLN 99. I will find a better chair that someone will sell for PLN 99, I will buy it cheaper, I don't know, from another source.* (Krakow, GP1)

### Opinions about the impact of transparency laws on doctors’ integrity and patients’ trust

As was the case during discussions about limiting the value of gifts, the problem of patients’ trust was raised during students’ group discussions about the notion of introducing compulsory transparency. Respondents often said that a result of such laws would be an end to speculation about what doctors obtain from industry. Full disclosure would end suspicions and unfounded accusations of corruption.

*M1 (III) Everything is transparent there. It does not give the patient room for speculation, and these speculations would always be pessimistic*.

*F1 (III) Worse.* (Krakow, GP1).

In one of the groups, participants concluded that such disclosure make a doctor seem more reliable to their patients. There is nothing to hide. It was also said that it should prevent the development of conspiracy theories surrounding relationships between doctors and the medical industry. There was no agreement among students whether patients would actually check it.

*F1(III) But I think that most of the patients would not check it anyway*.

*M1(III) Yes*.

*F1 (III) It would create an opportunity, but in practice no one would do it.*

*M1 (III) But the awareness that one* [a patient] *could verify could have an effect…*

*F1(III) This is already good.*

*M1 (III) It adds trust.* (Krakow, GP1)

Some students also expressed the opinion that such laws discourage the acceptance of gifts from pharmaceutical companies. Physicians may believe that openness about what they receive could negatively affect their image and for this reason they may opt out of accepting gifts.

*F1 (III) From the other side, if everyone could see the benefits for doctors, they* [the doctors] *may limit themselves too.*

*F2 (III) Yes, it could be so.*

*F1 (III) Because it is different when people can see…* (Krakow, GP2)

Students voiced the opinion that publicly available transparency registers would allow doctors to compare themselves with others, and that this might affect their notions of what is acceptable when cooperating with industry and what the limits are.

*M (III): The matter is left to the discretion of the doctor their conscience, and then they are given the opportunity to compare with others so that they can correct their view of what to take and what not to take.* (Krakow, GP3)

On the other side, some students believed that greater transparency concerning the extent of medical professionals’ cooperation with the pharmaceutical industry could not only stigmatize individual doctors but that it could also harm the image of the profession itself.

*F (IV) I wanted to say, that maybe it is better for Poles to not know about these gifts. Some of them may not be aware of this: that the gifts are given and received, and if they knew, it could result in even lower respect for the profession.* (Warsaw, GP1)

So, while transparency can strengthen patients’ trust in physicians because all significant benefits that physicians receive are declared, it also has the potential to undermine the credibility of physicians. When a patient learns that a physician is cooperating with a company that produces a drug prescribed for them, they may think that the drug is not necessarily the best one for them, and that it is only being prescribed because the physician has received benefits from the company.

*M (IV) A patient could become suspicious about a doctor. For example, if they prescribed a given drug indicated as being the best in a given situation, then the patient might not want to take it or he could go to another doctor because he might think that the doctor prescribed this medicine because they were given a gift…* (Warsaw, GP3)

Because transparency might undermine patients’ perceptions of physicians’ competence, physicians lose their freedom to choose to receive benefits from pharmaceutical companies. They are limited in their ability to cooperate and earn.

Some students expressed the view that, in the presence of transparency laws, accepting gifts is no longer neutral, but is stigmatized. Many students believed this is not good because it also applies to gifts that a physician accepts that will help their patients, or for purely educational purposes, and transparency laws may discourage physicians from accepting these gifts too. Here, one person pointed out that transparency laws change the tone of the morality of accepting gifts.

*F(II) Because it is published, it* [every gift] *is viewed as maybe not morally bad, but certainly not entirely good. If a man has to explain something as if he could potentially be ashamed of the fact that it is public, then it has a different tone right away.* (Gdansk, GP3)

During the discussion on the voluntary Transparency Code in force in Poland the students in this group expressed the idea that a voluntary solution “stigmatizes” doctors who agree to the publication of their dealings with companies.

*M (VI) Because it is, at the moment, a kind of unequal treatment: one will agree, another will not, and it will seem as though only a few doctors take these awards…, and the rest are clean. So* [as patient]*, I will go to those who are clean.* (Warsaw, GP2)

### Barriers to introducing a sunshine law in Poland

When deliberating about the possibility of introducing a Sunshine Law in Poland, students exchanged their views on potential barriers. First, many students thought that the Polish medical community would not agree to such a law and that practicing physicians would be unhappy. Historically, Polish healthcare has been underfunded, and physicians have become accustomed to the idea that gifts from industry are a supplement to their salary.

*F (V) And, for example, in Poland, each doctor, regardless of how much they earn, is still convinced that they do not earn enough and that there are many people in western countries who get much more…. And these employees fill the gap by simply interacting with pharmaceutical companies.* (Gdansk, GP2)

Second, the structure of the Polish health system results in patients having little flexibility when choosing a healthcare provider and providing patients with access to a database under a sunshine law might result in them deciding not to visit a physician, thereby harming their health.

M (V) *(…) If a patient had a look and saw that he or she* [their physician] *received something and decided not to go, another person would immediately take their place. Because of the waiting time, there will always be someone who will say, this it is not important to me. So, for me, it would have no impact on* [the number of] *patients waiting to see a doctor.* [But] *this would be bad for patients, waiting for a year to see another doctor…* (Gdansk, GP2)

Third, some students thought that Polish society is not ready for such a law. They thought that it would not completely resolve problems of patients losing trust in physicians because patients would suspect that physicians had accepted gifts in an informal manner.

*M1 (V) I know that in Poland it would not work, because of our society. Because everyone would think …this doctor…accepted this, this, this and that (…)*

*M2 (VI) Or they would say, that… the information was not made public.*

*F (IV) Indeed! It could be that somebody is trying to hide something and take things under the table (…)*. (Warsaw, GP2)

In addition, in many groups, students wondered whether patients would actually take the opportunity to check-up on the gifts obtained by physicians. But opinions here were usually divided. There were also doubts as to how the information would be used in Poland, it often being argued that perhaps it would result in attacks on physicians.

*F (IV) Battue would be next, all media would talk about it: television, radio, and the Internet would be buzzing. And they would probably get some individual saying what well-known doctors had taken, and when ...*

*M (V) 25 years ago they accepted this.* (Warsaw, GP1)

### Opposing views of students

Interestingly, the focus group conversations often reflected key opposing positions that are present in the public debate about relationships between the medical community and the pharmaceutical industry. While these positions were often taken by different groups of people within groups, sometimes a single person would take a position contrary to the rest of the group, depending on the context. These key opposing positions are presented in Table 1.

**Table 1:** Opposing views of students

Opposing views			
Issue		1	2
1	Opinions about the impact of legal restrictions on the value of gifts on patients’ trust	Legal limits could increase trust, because patients would know that small gifts are not a form of corruption	Some patients would be suspicious anyway, because they would think that doctors were not obeying the law or that even a gift of only 100 PLN in value is inappropriate
2	Opinions about the impact of legal restrictions on the value of gifts on physicians’ willingness to accept gifts	Legal regulation of the maximum value of gifts legitimizes accepting gifts per se (accepting something lawful is morally justified, and the law dispels moral doubts)	The introduction of a legal limit suggests that a gift above a certain value may have an undue influence (which in turn raises the issue of arbitrariness of the limit introduced)
3	Opinions about potential influence of small gifts on physicians	Because of their low value, small gifts cannot influence physicians, and, even if they do, any influence is not harmful to patients (the opinion of the majority)	Because the limit of 100 PLN is arbitrary and all gifts are given to affect physicians’ prescribing habits, to remain impartial, doctors should not accept gifts at all (a minority view)
4	General opinions on the rationale for disclosure of financial relationships	Financial relationships between companies and physicians should be disclosed because there is nothing to hide: nothing to be ashamed of	Mandatory disclosure stigmatizes relationships between the pharmaceutical industry and physicians (it encourages the impression that these relationships are something that physicians need to give excuses for)
5	The predicted influence of transparency on patients’ trust (if mandatory disclosure were to be introduced)	Patients would check which physicians received which gifts and they would stop fantasizing about corruption: trust should increase	Some patients would be suspicious anyway, and due to excessive, irrational loss of trust, patients could be harmed: they would take longer to obtain treatment, looking for a "clean" physician, or they would not take a medicine prescribed for them by a physician who declared a financial relationship with a pharmaceutical company – trust could decrease
6	Predictions about the impact of mandatory disclosure on the frequency with which physicians receive benefits	Mandatory disclosure would normalize receiving benefits as physicians would be able to see what is commonly accepted and what is inappropriate	Obligatory disclosure would discourage the receipt of benefits because the very awareness that it could be revealed (i.e., that checks could be made, even if nobody actually does so) would affect a physician's behavior (this again implies that there is something wrong with receiving benefits, or at least that it is seen as something wrong)
7	Predictions of the popularity of using transparency registers (if mandatory disclosure were to be introduced)	There is no point in introducing mandatory transparency registers because nobody will use them anyway	Out of curiosity and envy, every patient would check and there would be smear campaigns against physicians who are well paid by industry

## Discussion

In starting to discuss the current observations, it is worth outlining some of the historical, social, and cultural conditions of Polish medical practice. After World War II, the Polish state health service operated under the Siemaszko model. The socialist state (the Polish People's Republic) was responsible for ensuring all of society’s needs, including people’s health needs. During this time, government propaganda spoke of widely available and free health care, but in practice this system had numerous disadvantages. For example, there was high wastage of resources, services were of low quality, and some medicaments were unavailable. There were also staff shortages and the remuneration of medical workers was low. This led to corruption—especially gift-giving from patients (Sagan et al. 2011). After the collapse of the socialist system in 1989, Poland underwent a socioeconomic transformation, and, while these changes improved the quality of health services, they also led to greater inequalities in access to healthcare.^7^

When discussion of the consequences of pharmaceutical marketing being targeted at doctors began in the U.S. during the 1990s, the Polish market was just opening up to the foreign pharmaceutical industry and its advertising methods. It was not until 1993 that advertising of over-the-counter drugs to consumers was allowed (Polish Journal of Laws of 1993 No. 47, item 211, article 29). Lucrative and intensive relationships between physicians and PSRs flourished in the, still legally unregulated, environment of the 1990s (Polak 2011). This phenomenon began to be noticed at the beginning of the twenty-first century, not least because media reports about exclusive trips and luxurious gifts given to doctors aroused social controversy (Grzeszak 2000; Michalak and Romanowska 2001). Also of importance during this period was the fact that a significant reduction in corruption in many areas of Polish social life was achieved. In the following years, there was an increased awareness of the problem in the Polish medical community (Pasierski et al. 2003), and doctors’ relationships with PSRs are now regulated by law and by industrial and medical codes of ethics. Despite these new regulations, the occurrence of several conferences about the problem in recent years, and doctors being encouraged to disclose their relationships with the industry under the voluntary Transparency Code of the INFARMA Employers’ Union of Innovative Pharmaceutical Companies, the issue of conflicts of interest has never entered the mainstream of bioethical public debate in Poland. This is one reason why the presently observed low awareness of these issues among Polish medical students is not surprising.

The current study suggests that issues surrounding relationships between physicians and PSRs are rarely, if ever, covered during Polish medical education. Students were unaware, or at best barely aware, of current legal regulations concerning relationships with the pharmaceutical industry. These observations are consistent with previous quantitative research (Makowska et al. 2017). Although every medical university in Poland runs a medical ethics course, the issue of conflicts of interest is usually absent from syllabi. According to publicly available curricula, the most commonly considered bioethical issues are: abortion, euthanasia, patient rights, and transplantation. There are several potential reasons that conflicts of interest are so rarely discussed. As previously mentioned, this topic has never entered the mainstream of bioethical public debate in Poland, which is dominated by issues surrounding reproductive ethics.^8^ Furthermore, medical studies programs are crammed with content, and this limits the time available for classes in medical ethics. Also, it is likely that the issue of doctors’ relationships with PSRs is far from the top of the priority list of medical ethics lecturers.^9^ Polish medical students also have classes in medical law (which are sometimes connected with courses in medical ethics or forensic medicine, but also sometimes separate), but such classes also involve topics that are deemed more important than conflicts of interest. However, having said all this, the possibility that the currently discussed legal issues were mentioned during some lectures cannot be ruled out: it is possible that students participating in the focus groups did not consider these issues important or interesting enough for them spring to mind.

Another hypothetical reason why legal awareness among medical students may be so low is related to much deeper, structural causes. Public opinion polling data show that the Polish people generally tend to have rather low respect for the law (CBOS 2013). This situation is most commonly explained by historical factors (Kuisz 2018), in particular, the long periods of time where laws have been imposed by foreign powers (e.g., the partitions of Poland which happened toward the end of the eighteenth century, and the periods of Nazi occupation and Soviet domination which occurred during the twentieth century). This phenomenon is likely to have consequences for the future development of bioethics in Poland since bioethical issues are by their very nature often related to legal regulation, societies and legislators having to decide when and where to impose limitations on different branches of modern medicine and technology.^10^

During students’ medical studies, significant parts of their developing knowledge, skills and attitudes are transmitted outside the lecture hall, mainly by observing practicing doctors at work. However, our participants’ responses gave the impression that their experiences during internships also failed to provide them with knowledge or strong convictions as to which behaviors in their relationships with PSRs might be considered appropriate. This lack of knowledge and students’ feelings of uncertainty as to which behaviors are legally and morally permissible need to be remedied to prepare students better for their future profession.

Importantly, none of the groups’ discussions about the law led to deeper consideration of how creating new laws forces more ethical marketing behavior on the pharmaceutical industry, and what implications this can have for the wider public health. Nevertheless, students often drew attention to the important topic of patient trust, which is crucial both for medical outcomes and the physician–patient relationship (Bonds et al. 2004; Kaczmarek 2019). Their discussions highlighted an ambivalent relationship between disclosure and trust.

This ambivalent relationship seems relevant and peculiar in the context of the Polish Code of Medical Ethics (2014),^11^ which, among other things, regulates physicians’ interactions with the medical industry. Thus, Article 51a.1 states: “*The physician should not accept benefits from representatives of the medical industry if this could influence the objectivity of their professional judgment or undermine trust in the medical profession”.* As can be seen from Table 1, it is unclear whether limiting the value of PSRs’ gifts guarantees full preservation of trust, as some students thought that people could consider any gift to be inappropriate because of these offerings’ association with marketing. Similarly, there was no consensus about the rationale for disclosing financial relationships with the pharmaceutical industry: should they be disclosed because there is nothing to be ashamed of, or, on the contrary, does disclosure imply stigmatization of such relationships by giving the impression that they are something that physicians need to provide excuses for? Some participants expressed worries that people’s awareness of a conflict of interest may result in an excessive loss of trust, and therefore harm patients. For example, a patient could experience negative consequences if they refused to be vaccinated because they saw a pen with the logo of the vaccine’s producer in their doctor’s office. However, it is difficult to specify the point at which loss of trust due to conflicts of interest becomes unacceptable, although it is reasonable to expect that physicians should gain their knowledge about new drugs mainly from independent, impartial, scientific resources, and not mainly from PSRs (who are often educated in marketing rather than pharmacy or medicine). Impartiality seems to be one major condition of trustworthiness, and, by definition, conflicts of interest constitute a risk to impartiality. For all these reasons, we argue that Article 51a.1 should be reconsidered, because in its current form it fails to provide any clear directives for doctors’ behavior.

More research is needed to ascertain whether disclosure might increase bias in advice of a person who discloses conflicts of interest, especially given reports of the so called “perverse effects of disclosing conflicts of interest” (Cain et al. 2005) and how does the disclosure really influence trust of the patients.

Although most participants seemed to know that relationships with PSRs are of a marketing nature, it was worrying to observe that they were unaware of research suggesting that even the provision of small benefits (e.g., sponsored meals or pens; DeJong et al. 2016; Katz et al. 2010) has been shown to be associated with the making of decisions coherent with a sponsor’s interests (e.g., more frequent prescribing of branded drugs instead of generics; Yeh et al. 2016). Students seemed to underestimate the strength of the principle of reciprocity in marketing.

The above said, students were aware of the fact that the pharmaceutical industry provides physicians with things that are underfunded by the public healthcare system—both basic goods, such as medical tools and office supplies, and less basic, but equally essential, things such as opportunities to learn (e.g., the covering of conference fees, which is particularly important in the context of physicians’ legal obligation to continue their professional education once practicing). In the eyes of some students, the lack of these things seemed to be more scandalous than any possibility of the pharmaceutical industry having an undue influence. Such opinions coincide with one of the key arguments used by the Polish medical community to justify lucrative relationships with the pharmaceutical industry. However, such arguments may be coming less tenable since Polish physicians are now receiving better remuneration than was the case even just a few years ago (Głowny Urząd Statystyczny 2012; Głowny Urząd Statystyczny 2018), and national spending on healthcare is to be increased (IQVIA 2018). Further research is needed to determine whether these changes have an impact on Polish physicians’ willingness to accept benefits from the industry and their willingness to disclose these benefits.

Moreover, it is worth noting that the Polish medical community is strictly hierarchical (Sokołowska 1986; Hartman 2012) and students occupy a very low position in this hierarchy. Also, some lecturers accept benefits from industry. Therefore, for a student to raise their moral doubts about current practices and make a public declaration that physicians should not accept gifts from pharmaceutical companies requires self-confidence and courage. This is even more true because in Poland there is a lack of strong public or student organizations speaking out in favor of transparency in medicine (although a group called No Free Lunch Poland exists, it is still young, little known and organizationally weak).

There is a lack of research in Poland regarding both medical community –pharmaceutical company contacts and the presence of conflicts of interest in the educational policies of medical schools. In our opinion current self-regulation policies on transparency between the pharmaceutical industry and medical professions in Poland are insufficient and they do not perform their function well. In their current form, transparency reports do not allow anybody (e.g., patients, researchers or journalists) to check whether a specific physician or key opinion leader has financial ties with a given company, because data are voluntarily provided and difficult to manage. Yet, Polish physicians are resistant to more clarity being provided: according to INFARMA in 2016, 78% of physicians did not agree that the details of the benefits they receive should be published. Despite this, change is needed, not least because the current self-regulation policies are unfair on those physicians who decide to disclose their data, and they give an unfair advantage to companies which do not participate in self-regulation.

In our opinion, introducing legal regulations at the European level should be considered. Many companies have obliged themselves to disclose payments given to healthcare professionals, healthcare organizations and patient organizations (see, e.g., the EFPIA Code of Practice 2019). European legal regulations could harmonize international practice and minimize bureaucracy.

Even a combination of legal regulations restricting the value of gifts and introducing transparency will not solve all the current problems because the diminution in trust in medicine caused by conflicts of interest will not be ameliorated. We should try to identify the most effective alternatives for eliminating the negative impact of industrial marketing on the knowledge, attitudes and actions of physicians. Among other things, these alternatives might take the form of educating medical students about conflicts of interest, minimalizing meetings with PSRs, the licensing of PSRs, and academic detailing (meetings with scientific professionals not connected with pharmaceutical industry). Further public debate and debate within the medical community is needed with respect to these issues.

This study has limitations which are discussed in detail in another article stemming from the present research project (Makowska 2021). Here, we will confine ourselves to mentioning issues that might have influenced the dynamics and discussions of focus groups: (1) the time frame of the research meant that the selection of respondents was based solely on their availability, and as a result a preponderance of third year students took part in the research; (2) the first three groups were slightly larger than the others; (3) some respondents did not know each other; this is difficult to avoid when communities of people are being studied (Krueger and Casey 2014).

## Conclusions

The currently presented qualitative research is the first of its kind in Poland. It also fills a gap in international research regarding medical students’ opinions of legal regulations surrounding physicians’ cooperation with the medical industry.

The main points uncovered by the study are: (1) the students seemed to be unaware of the existence of the relevant regulations, this suggesting that coverage of the regulations was probably absent from their curriculum: this should raise concerns as the lack of knowledge identified is likely to lead to uncertainty in students as to which behaviors are legally and morally permissible; (2) a particularly popular view was that less valuable gifts, such as those up to a value of PLN 100, should not trigger a need for reciprocity—a desire to repay a company by prescribing their products; (3) students perceive an ambivalent relationship between disclosure and trust: this requires more in-depth research and bioethical reflection; (4) the students thought that the Polish medical community would not be open to the introduction of transparency laws and the casting of light upon the benefits its members receive; this suggests that further public debate is needed on these issues.

## Data Availability

The interview scenario and a transcription of all data will be in the public repository of the Social Data Repository: Repozytorium Danych Społecznych.

## Footnotes

1 The study was conducted on a sample of 553 medical students from the Medical University of Warsaw, 224 of whom were in their final (6th) year.

2 The limit of PLN 100 was added in a 2007 amendment, previously the term “negligible” was used.

3 A Polish transcription of the interviews are publicly available at… [10.18150/UFEA7T].

4 In Poland medical studies last 6 years.

5 *F* female, *M* male. The Roman numerals in parentheses indicate a student’s year of study. GP – group followed by its number. Text in square brackets [] has been added by the authors to facilitate understanding.

6 This quotation has also been mentioned in another article stemming from the present research project (Makowska 2021).

7 There is currently a Polish public healthcare system under which patients’ access to medical services is free at the point of delivery, but this is currently underfunded and waiting periods are often very long (they can last months or years); there is also a private sector where people can pay directly for treatment and where waiting times are much shorter, but this type of care is only available to the richer part of Polish society.

8 In Poland, public debates on bioethics are strongly dominated by the issue of abortion. This topic is constantly debated due to several changes in abortion laws that have occurred since the political and economic transformation of the 1990s. Poland has some of the strictest abortion laws in Europe because of the strong influence of the Catholic Church. The most recent legal change took place in 2020 when the Constitutional Court ruled abortion on the grounds of incurable, even lethal, defects of a fetus to be unconstitutional, this leading to mass protests and strikes in the middle of the COVID-19 pandemic.

9 At Polish medical universities, classes in medical ethics are mostly taught by physicians and philosophers. Although Poland has strong traditions in the philosophy of medicine and ethics (mainly virtue ethics), the profession of bioethicist, in the Western sense, is rather new, and the possibility of obtaining a master’s degree in bioethics has only emerged in the past few years.

10 The relationship between law and ethics is a complex philosophical problem, and unfortunately there is not sufficient space to provide a detailed analysis of the issues involved here.

11 The Polish Code of Medical Ethics is not legally binding, but Polish physicians are obliged to abide by its rules. Under the jurisdiction of the Court of The Polish Chamber of Physicians and Dentists, doctors may face consequences (as severe as losing the right to practice their profession) for breaches of the Code. The Code is highly demanding in that it establishes the obligations of a doctor that occur in ideal circumstances: obligations which are often difficult to fulfill in everyday practice (e.g., it is difficult to preserve a patient’s right to privacy in an extremely overcrowded hospital ward). Thus, physicians’ actual practices often deviate widely from the Code’s specifications, and doctors rarely face consequences for breaches of the Code (these usually only occurring in cases of severe misconduct).

## References

[CR1] Austad KE, Avorn J, Franklin JM, Kowal MK, Campbell EG, Kesselheim AS (2013). Changing interactions between physician trainees and the pharmaceutical industry: A national survey. Journal of General Internal Medicine.

[CR2] Austad KE, Avorn J, Kesselheim AS (2011). Medical students' exposure to and attitudes about the pharmaceutical industry: A systematic review. PLoS Medicine.

[CR3] Berry, L.L. 1983. Relationship marketing. In *Emerging perspectives of services marketing*, ed. L. L. Berry, L. Shostack, and G. Upah. Chicago: American Marketing Association, 25–80.

[CR4] Bonds DE, Camacho F, Bell RA, Duren-Winfield VT, Anderson RT, Goff DC (2004). The association of patient trust and self-care among patients with diabetes mellitus. BMC Family Practice.

[CR5] Braun V, Clarke V (2006). Using thematic analysis in psychology. Qualitative Research in Psychology.

[CR6] Cain DM, Loewenstein G, Moore DA (2005). The dirt on coming clean: Perverse effects of disclosing conflicts of interest. The Journal of Legal Studies.

[CR7] CBOS [Public Opinion Research Center]. 2013. O przestrzeganiu prawa i funkcjonowaniu wymiaru sprawiedliwości w Polsce [On compliance with the law and the functioning of the justice system in Poland] Warsaw: CBOS Research Announcement No 5.

[CR8] Choudhry NK, Stelfox HT, Detsky AS (2002). Relationships between authors of clinical practice guidelines and the pharmaceutical industry. Journal of American Medical Association.

[CR9] David C, Mansfield P (2010). What do medical students think about pharmaceutical promotion?. Australian Medical Student Journal.

[CR10] DeJong C, Aguilar T, Tseng CW, Lin GA, Boscardin WJ, Dudley RA (2016). Pharmaceutical industry–sponsored meals and physician prescribing patterns for medicare beneficiaries. JAMA Internal Medicine.

[CR11] EFPIA - European Federation of Pharmaceutical Industry Associations. (2019). EFPIA Code of Practice.

[CR12] Fickweiler F, Fickweiler W, Urbach E (2017). Interactions between physicians and the pharmaceutical industry generally and sales representatives specifically and their association with physicians’ attitudes and prescribing habits: A systematic review. British Medical Journal Open.

[CR13] Fitz, M. M., Homan, D., Reddy, S., Griffith III, C. H., Baker, E., & Simpson, K. P. 2007.The Hidden Curriculum: Medical Students’ Changing Opinions toward the Pharmaceutical Industry. Academic Medicine, 82(10Suppl), S1–3. 10.1097/ACM.0b013e31813e7f02. 10.1097/ACM.0b013e31813e7f02 17895670

[CR14] Flick U (2018). Designing qualitative research.

[CR15] Główny Urząd Statystyczny [Central Statistical Office]. 2012. Struktura wynagodzeń według zawodów w październiku 2010 roku [Pay structures by occupation in October 2010]. https://stat.gov.pl/obszary-tematyczne/rynek-pracy/pracujacy-zatrudnieni-wynagrodzenia-koszty-pracy/struktura-wynagrodzen-wedlug-zawodow-w-pazdzierniku-2010-r-,4,5.html. Accessed 9 Jan 2021.

[CR16] Główny Urząd Statystyczny [Central Statistical Office]. 2018. Struktura wynagrodzeń według zawodów w październiku 2016 [Pay structures by occupation in October 2016]. https://stat.gov.pl/files/gfx/portalinformacyjny/pl/defaultaktualnosci/5474/4/8/1/struktura_wynagrodzen_wedlug_zawodow_w_pazdzierniku_2016.pdf. Accessed 9 Jan 2021.

[CR17] Grabitz, P.R., Z. Friedmann, S. Gepp, L.U. Hess, L. Specht, M Struck, S. Tragert, T. Walther, and D. Klemperer. 2019. Conflict of Interest Policies at German medical schools - A long way to go. BioRxiv. 10.1101/809723. 10.1136/bmjopen-2020-039782 PMC7528426 32998930

[CR18] Grzeszak, A. 2000. Przychodzi rep do lekarza [The rep visits the doctor]. Polityka, 48, 30–32, December 16.

[CR19] Hartman J (2012). Bioetyka dla lekarzy. [Bioethics for physicians].

[CR20] Hodges BD (1995). Interactions with the pharmaceutical industry: Experiences and attitudes of psychiatry residents, interns and clerks. Canadian Medial Association Journal.

[CR21] INFARMA. 2015. Kodeks przejrzystości. [Transparency code]. https://www.kodeksprzejrzystosci.pl/assets/files/etyka/Kodeks_Przejrzystosci_wydanie_5.pdf. Accessed 9 Jan 2021.

[CR22] IQVIA (2018). Finansowanie ochrony zdrowia w kontekście efektów społeczno – gospodarczych. [Health care financing in the context of socio-economic effects].

[CR23] Kaczmarek E. 2019. Conflicts of interest, impartiality and trust. In *Shedding light on transparent cooperation in healthcare.**The way forward for sunshine and transparency laws across Europe*, ed. M. Rodzinka, M. Fallon-Kund & C. Marinetti. Brussels: Mental Health Europe, Europe, 52–55.

[CR45] Kaczmarek E, Makowska M (2020). Wymaganie obecności na zajęciach w czasie choroby jako przykład ukrytego programu studiowania medycyny. [Requiring class attendance during illnessas an example of the hidden curriculum in medical studies]. Annales. Ethics in Economic Life.

[CR24] Katz D, Caplan AL, Merz JF (2010). All gifts large and small: Toward an understanding of the ethics of pharmaceutical industry gift-giving. The American Journal of Bioethics.

[CR25] Krueger RA, Casey MA (2014). Focus groups: A practical guide for applied research.

[CR26] Kuisz J (2018). Koniec pokoleń podległości [The end of generations of subordination].

[CR27] Makowska, M. 2010. *Etyczne standardy marketingu farmaceutycznego. [Ethical standards of pharaceutical marketing].* Warsaw: CeDeWu.

[CR28] Makowska M (2017). Polish physicians’ cooperation with the pharmaceutical industry and its potential impact on public health. PloS one.

[CR46] Makowska M (2021). How polish medical students are socialised to cooperate with the pharmaceutical industry: a focus group study of the importance of informal, hidden and null curricula. Health Sociology Review (in-print).

[CR29] Makowska M, Sillup GP, Lee MJ (2017). Pharma’s marketing influence on medical students and the need for culturally competent and ctricter policy and educational curriculum in medical schools: A comparative analysis of social scientific research between Poland and the US. The Journal of Healthcare Ethics and Administration.

[CR30] Michalak J, Romanowska D (2001). Chorzy lekarze [Sick doctors]. Wprost.

[CR31] Naczelna Izba Lekarska [Polish Supreme Medical Chamber]. 2014. Kodeks etyki lekarskiej [Code of Medical Ethics]. https://nil.org.pl/uploaded_images/1574857770_kodeks-etyki-lekarskiej.pdf. Accessed 9 Jan 2021

[CR32] Pasierski T, Pińkas J, Religa Z (2003). Czy możliwa jest etyczna współpraca między lekarzami a firmami farmaceutycznymi [Is ethical cooperation between doctors and pharmaceutical companies possible?]. Standardy Medyczne.

[CR33] Polak P (2011). Nowe formy korupcji. Analiza socjologiczna sektora farmaceutycznego w Polsce [New forms of corruption. A sociological analysis of the pharmaceutical sector in Poland].

[CR34] Polish Journal of Laws of 1993 No. 47, item 211, On combating unfair competition.

[CR35] Polish Journal of Laws of 2001 No. 126, item 1381, Pharmaceutical law.

[CR36] Polish Journal of Laws of 2008 No. 210, item 1327, Minister of Health Regulation on advertising of medicinal products.

[CR37] Polski Związek Pracodawców Przemysłu Farmaceutycznego [Polish Association of Pharmaceutical Industry Employers]. (2017). Zasady i wymagania dotyczące przejrzystości. [Rules and requirements for transparency]. http://www.producencilekow.pl/wp-content/uploads/2017/11/transfer-korzysci.pdf. Accessed 9 Jan 2021.

[CR38] Sagan A, Panteli D, Borkowski W, Dmowski M, Domański F, Czyżewski M, Goryński P, Karpacka D, Kiersztyn E, Kowalska I, Księżak M, Kuszewski K, Leśniewska A, Lipska I, Maciąg R, Madowicz J, Mądra A, Marek M, Mokrzycka A, Poznański D, Sobczak A, Sowada C, Świderek M, Terka A, Trzeciak P, Wiktorzak K, Włodarczyk C, Wojtyniak B, Wrześniewska-Wal I, Zelwiańska D, Busse R (2011). Poland: Health system review. Health Systems in Transition.

[CR39] Sah S, Fugh-Berman A (2013). Physicians under the influence: Social psychology and industry marketing strategies. The Journal of Law, Medicine & Ethics.

[CR40] Scheffer P, Guy-Coichard C, Outh-Gauer D, Calet-Froissart Z, Boursier M, Mintzes B, Borde JS (2017). Conflict of interest policies at French Medical Schools: Starting from the bottom. PLoS ONE.

[CR41] Sierles FS, Brodkey AC, Cleary LM, McCurdy FA, Mintz M, Frank J, Lynn J, Chao J, Morgenstern Z, Shore W, Woodard JL (2005). Medical students' exposure to and attitudes about drug company interactions: A national survey. Journal of American Medical Association.

[CR42] Sokołowska M (1986). Socjologia medycyny. [Sociology of medicine].

[CR43] Van Harrison R (2003). The uncertain future of continuing medical education: Commercialism and shifts in funding. Journal of Continuing Education in the Health Professions.

[CR44] Yeh JS, Franklin JM, Avorn J, Landon J, Kesselheim AS (2016). Association of industry payments to physicians with the prescribing of brand-name statins in Massachusetts. JAMA Internal Medicine.

